# A Brazilian Portuguese translation, cultural adaptation and validation of the Arrhythmia-Specific questionnaire in Tachycardia and Arrhythmia (ASTA) health-related quality of life (HRQOL) scale

**DOI:** 10.1371/journal.pone.0256851

**Published:** 2021-08-27

**Authors:** Priscila Moreno Sperling Cannavan, Fernando Piza de Souza Cannavan, Henrique Ceretta Oliveira, Ulla Walfridsson, Maria Helena Baena de Moraes Lopes

**Affiliations:** 1 School of Nursing, University of Campinas, Unicamp, Campinas, São Paulo, Brazil; 2 Department of Clinical Medicine, Discipline of Cardiology, School of Medical Sciences, University of Campinas, Unicamp, Campinas, São Paulo, Brazil; 3 Department of Cardiology, University Hospital, Linköping, Sweden; 4 Department of Medical and Health Sciences, Linköping University, Linköping, Sweden; Faculty of Health Sciences - Universidade da Beira Interior, PORTUGAL

## Abstract

**Introduction:**

The health-related quality of life (HRQOL) of patients with tachyarrhythmia can be negatively influenced by the clinical manifestations. The evaluation of HRQOL with validated instruments can provide valuable information that will contribute to clinical decision-making and treatment. In Brazil, however, there is no available scale that evaluates HRQOL in different types of arrhythmia. The purpose of this study was to adapt the Arrhythmia-Specific Questionnaire in Tachycardia and Arrhythmia-HRQOL scale (ASTA-HRQOL scale) to the Brazilian culture, and to assess the psychometric properties of the adapted questionnaire.

**Methods:**

The study used a methodological process of cultural adaptation based on international literature guidelines. The analyses were performed with 172 participants, 32 for cultural adaptation and 140 for psychometric validation. Calculation included analysis of reliability by Cronbach’s α coefficient, construct validity with convergent validity using the WHOQOL-BREF questionnaire and by the Spearman correlation coefficient, Average Variance Extracted, and assessment of confirmatory factor analysis.

**Results:**

The translation and adaptation processes showed a satisfactory degree of comprehension and applicability (93% reported them to be easy to understand). Confirmatory factor analysis indicated exclusion of one item from the mental scale, but after qualitative analysis the item was retained. The items presented adequate internal consistency (Cronbach’s alpha coefficient = 0.88), and an inverse correlation of moderate magnitude with the physical domain (rho = -0.63) and with the mental domain (rho = -0.58) of the WHOQOL-BREF.

**Conclusions:**

The Brazilian Portuguese version of the ASTA-HRQOL scale, the ASTA-Br-HRQOL scale, can be a valuable tool for use in clinical practice and research.

## Introduction

Health-related quality of life (HRQOL) has been extensively studied and is used to describe how different types of diseases and treatments can influence patients’ daily lives [1]. The evaluation of HRQOL implies contemplating the dimensions of individuals’ lives, discriminating among the physical, social and emotional domains [2].

The HRQOL of patients with tachyarrhythmia can be significantly and negatively affected due to the manifestations, whether physical or emotional, affecting the performance of the usual activities of daily living [3–7]. Therefore, HRQOL measurement is an important way to evaluate and describe the life situation of these patients [8].

To properly evaluate HRQOL, a reliable and valid instrument is required. However, most of the instruments for this assessment in patients with arrhythmias are specifically aimed at individuals with atrial fibrillation (AF) [9–14].

In order to evaluate HRQOL in patients with different forms of tachyarrhythmia, the Arrhythmia-Specific questionnaire in Tachycardia and Arrhythmia-HRQOL (ASTA-HRQOL) scale was developed and validated, being the only validated questionnaire evaluating patients with supraventricular and ventricular arrhythmias, allowing comparison between patients with different types of arrhythmias [15, 16].

The original ASTA-HRQOL scale had its internal consistency evaluated by Cronbach’s α coefficient. Construct validity was evaluated using item-total correlations, convergent and discriminant validity, and confirmatory factor analysis, showing satisfactory results [15].

Since there is no similar scale in Brazil that evaluates HRQOL in different forms of tachyarrhythmia, the purpose of this study was to culturally adapt and assess the psychometric properties of the Brazilian version of the ASTA-HRQOL scale, the ASTA–Br-HRQOL scale, in a Brazilian group of patients with cardiac arrhythmia.

## Methods

### Study design and population

This was a methodological study of a quantitative approach [17], carried out from May to October 2018 in an outpatient clinic of a public university hospital and in a private clinic, both specialized in the care of patients with cardiac arrhythmias and located in the state of São Paulo, Brazil. Participants aged ≥18 years, without implantable electronic cardiac devices and diagnosed with tachyarrhythmia for more than three months, were included in the study.

### Informed consent and ethical considerations

After obtaining permission from the original developer, the ASTA questionnaire was translated and adapted into a Brazilian Portuguese version and the Research Ethics Committee of the University of Campinas–Brazil, approved the research (CAAE 78539617.7.0000.5404). All the participants signed an informed consent form before the interview.

### ASTA-HRQOL scale

The Arrhythmia-Specific questionnaire in Tachycardia and Arrhythmia (ASTA) is divided into separate parts for assessing symptoms and HRQOL in connection with different forms of arrhythmia [15, 18]. These parts of the questionnaire can be applied together or separately. The focus of the present study was the cultural adaptation and validation of the ASTA-HRQOL scale, the ASTA-Br-HRQOL scale. The Brazilian Portuguese version of the ASTA questionnaire focusing on symptoms has recently been validated [19].

The ASTA-HRQOL scale has 13 items covering a physical and mental domain, with responses varying from 0 ("no") to 3 ("yes, a lot") and scores varying from 0 to 39, which can be recalculated with a syntax, giving scores ranging between 0 to 100, with lower scores indicating better HRQOL related to tachyarrhythmia [15]. Please see S1 Appendix for the 13 questions.

### Procedure

The study was carried out in three stages: translation and cultural adaptation of the instrument, pre-test and validation. After obtaining permission from the original developer to use and translate the ASTA questionnaire, the methodological process of cultural adaptation was conducted according to the guidelines recommended by Beaton et al. [20], including the following stages: translation, synthesis of translations, backward translation, review of an expert committee and pre-test.

The initial stage was the translation of the instrument from the Swedish language into the Brazilian Portuguese language. Two independent translators performed this procedure, with one translator being informed of the subject of the study and the other translator not informed. The versions were compared by the researcher and by a mediator, in an effort to identify and settle the divergences, resulting in the synthesis of the translations.

From the synthesis of the translations, the questionnaire was backward translated into the original Swedish language. This step was important for verifying that the translated version reflected the same items contained in the original version. This process was carried out by two independent translators who had Swedish as their native language [21].

Subsequently, for the validation of the content and to resolve the discrepancies, a committee of experts was formed, comprising eight members: a methodologist (nurse practitioner with knowledge of the theoretical framework), a linguist with knowledge of the Portuguese and Swedish languages, a physician specialist in arrhythmias from the Brazilian Society of Cardiac Arrhythmias, a Brazilian nurse with clinical experience in cardiology in Sweden, and the four translators who carried out the revision of the previous versions.

At this stage, the Content Validity Index (CVI) was used to identify semantic, idiomatic, cultural and conceptual equivalence between the original and translated scale [22].

The CVI assessed the agreement between the judges [22] and was calculated on the basis of the expert judges’ assessment. For the calculation of the CVI, each judge analyzed the scale items from the four equivalences (semantic, idiomatic, cultural and conceptual), using a Likert scale ranging from one to four, one representing "not equivalent"; two, "impossible to evaluate without revision"; three, "equivalent, but some changes are needed" and four, "absolutely equivalent". After the judges’ evaluation, the CVI was calculated as recommended in the literature [22]. A CVI ≥ 0.8 was considered acceptable, while items that had a CVI below 0.8 were reviewed. One item N0 7, had a CVI below 0.8 (0.74). Three other items were modified (5, 10 and 12) as suggested by the experts. Please see Table 1. At the end, the pre-final version of the instrument for the pre-test was developed.

**Table 1:** Comparisons between the syntheses of the translations presented to the experts and the final version of the ASTA-Br-HRQOL.

Question	Synthesis of Translations presented to the Experts	Final version in Portuguese approved by the Experts
Question 5	Você sente que sua capacidade física está prejudicada devido à sua arritmia? / Do you feel that your physical capacity is impaired due to your arrhythmia?	Por causa da sua arritmia cardíaca você acha que sua capacidade ou desempenho físico está prejudicado? / Because of your cardiac arrhythmia, do you think your physical capacity or performance is impaired?
Question 7	Sua arritmia cardíaca faz você se sentir deprimido ou triste? / Does your cardiac arrhythmia make you feel depressed or sad?	Por causa da sua arritmia cardíaca você se sente abatido ou triste? / Because of your cardiac arrhythmia do you feel dejected or sad?
Question 10	Você sente que sua arritmia cardíaca prejudica sua vida amorosa/sexual? / Do you think your cardiac arrhythmia damages your love / sex life?	Por causa da sua arritmia cardíaca você sente que a convivência com seu parceiro(a)/vida sexual está prejudicada? / Because of your cardiac arrhythmia do you feel that living with your partner / sex life is impaired?
Question 12	Sua arritmia cardíaca tem feito com que sua situação de vida piore? / Has your cardiac arrhythmia made your life worse?	Sua arritmia cardíaca faz com que sua qualidade de vida piore?/ Does your cardiac arrhythmia make your quality of life worse?

(Only changed questions are shown in this table)

The pre-test was the last stage of the cultural adaptation process. Ideally, it should be performed with 30 to 40 individuals from the targeted population [20]. After the adapted questionnaire was used, the Usability Evaluation of Instruments [23] was applied, checking the understanding of the instructions, questions and forms of response. After corrections, if necessary, the final version of the ASTA-Br-HRQOL, translated and adapted to a Portuguese context, was defined. Please see S2 Appendix.

### Questionnaires

The ASTA-Br-HRQOL scale was used along with the WHOQOL-BREF questionnaire.

The WHOQOL-BREF questionnaire assesses generic quality of life and HRQOL, and is the abbreviated version of the WHOQOL-100 [24], a questionnaire developed by the World Health Organization (WHO) Quality of Life group, adapted to Portuguese by Fleck (2000) [25], and composed of 26 questions, divided into four domains, including a physical and a mental domain.

### Data analysis

Descriptive analyses were performed for the categorical and continuous variables, referring to sociodemographic and clinical characterization. The ASTA-Br-HRQOL scale was psychometrically evaluated for construct validity and reliability. Internal consistency reliability was measured by Cronbach’s alpha coefficient [26] and composite reliability. Values ≥ 0.7 were considered satisfactory [27]. For convergent construct validity, the WHOQOL-BREF was used and evaluated by the Spearman correlation coefficient (rho) [28]. This coefficient varies from -1 to 1. A negative or inverse relationship between variables is indicated by values closer to -1, values close to 1 indicate a positive relationship, and values close to 0 indicate no correlation. Schober [29] suggests the following classification for the magnitude of the correlation: 0.00–0.10 negligible correlation, 0.10–0.39 weak correlation, 0.40–0.69 moderate correlation, 0.70–0.89 strong correlation and 0.90–1.00 very strong correlation.

The WHOQOL-BREF questionnaire was used for construct validity with evaluation of convergent validity by correlations between the ASTA-Br-HRQOL and the physical and mental domains of the WHOQOL-BREF questionnaire. The hypothesis was that the correlation between the ASTA-Br-HRQOL and the physical and mental domains of the WHOQOL-BREF questionnaire should be negative and moderate, since they measure the same concept, but with an inverse relationship between the scores, worst quality of life indicated by low Whoqol-bref scores and by high ASTA-BR-QVRS scores [15].

The construct validity was further evaluated by means of a second order confirmatory factor analysis (CFA). We used structural equation models using Partial Least Squares (PLS) as the estimation method [30]. The analysis of the factor model comprised two steps: analysis of the convergent and discriminant validity of the proposed model.

In the convergent validity analysis, the results of Average Variance Extracted (AVE) for each of the model factors were evaluated. Values of AVE higher than 0.5 indicated that the model converged satisfactorily [31]. The other criterion used to evaluate the convergent validity of the factor model was the factor load values obtained. Factor load values greater than or equal to 0.5 [32] were considered adequate.

The discriminant validity was initially evaluated using the Fornell-Larcker criterion [31]. This method compares the square root of the AVEs with the correlation values between the factors. The model has discriminant validity if the square roots of the AVEs are larger than the correlations between the factors. The other criterion considered to evaluate the discriminant validity was cross-loading analysis. In this case, we observed whether the factor load of a given item was higher in the factor in which it was initially allocated than in the other factors of the model.

To establish the sample size for instrument validation, the recommendations of Hair et al. [32] were considered for the confirmatory factor analysis, which suggests five to ten respondents for each item of the instrument. Considering ten respondents per item, it would be necessary to have a sample of at least 130 respondents. Ten patients were added to compensate for sample loss, and finally 140 patients were invited to participate in the study.

The data were collected and managed through the REDCap (*Research Electronic Data Capture*) [33] platform and then transferred to the Statistical Analysis System for Windows®, version 9.4, SPSS 22 and SmartPLS 3.2.1 software. A P-value of < .05 was considered significant.

## Results

### Cultural adaptation

The expert committee recommended modifications to improve the understanding of the scale and to facilitate its application, as shown in Table 1. It was also suggested to change the layout of the ASTA-Br-HRQOL in order to facilitate its introduction to the respondent.

For the pre-test, the instrument was administered to a sample of 32 individuals with characteristics similar to those for whom the instrument is intended. After the use of the ASTA-Br-HRQOL, the participants were asked about their understanding of the instructions, the questions, and how to answer them, through the Instrument Practicability Assessment questionnaire [23]. All participants reported that the instructions were easy to understand and they knew how they should respond. The majority (93%) reported that the items were easy to understand. The first two (6.2%) participants who answered the questionnaire had difficulty understanding the meaning of the terms "to a certain extent" and "quite a lot". Respondents suggested changing "to a certain extent" to "little" and "quite a lot" to "moderate." The difficulty in understanding the meaning of these words and the suggestions of these participants were brought to the knowledge of the committee of experts, who chose to replace the terms in question with those suggested by the participants.

### Psychometric validation

For validation, 140 patients who met the eligibility criteria for participating in the validation of the ASTA-Br-HRQOL scale were contacted before the medical arrhythmologist consultation. The researcher explained the research project and the patients signed the consent form. The interviews and data collection took place in a private room and the data were collected by the researcher using a tablet.

Table 2 presents the sociodemographic characteristics and Table 3 presents the clinical characteristics of the participants.

**Table 2:** Characteristics of the patients for the psychometric evaluation of the ASTA-Br-HRQOL scale (n = 140).

Age—years mean (SD)	55 (13.10)
Gender F/M (F %)	77/63 (55)
Cohabitant/living alone (cohabitant %)	124/16 (89)
Education (%)	
Non-formal education	10 (7)
Elementary school	87 (62)
High school	30 (22)
University degree	13 (9)
Occupation (%)	
Retired	60 (43)
Employed	45 (32)
Housewife	17 (12)
Sick leave	11 (8)
Unemployed	7 (5)
Remuneration (%)	
Once/twice the minimum wages *	111 (79)
Three/four times the minimum wages	18 (12)
More than four times the minimum wages	11 (8)

*minimum wages in force: R$ 954.00 or USD 257.88 or EUR 222.91

ASTA-Br-HRQOL scale: The Arrhythmia-Specific Questionnaire in Tachycardia and Arrhythmia—Brazilian version- health-related quality of life scale

**Table 3:** Clinical characteristics of the participants (n = 140).

Diagnoses	AF	AFL	AVNRT	WPW	PSVT	AT	VA
	n (%)	n (%)	n (%)	n (%)	n (%)	n (%)	n (%)
	71 (51)	19 (14)	16 (11)	14 (10)	7 (5)	2 (1)	11 (8)
**Treatments performed**							
Medication	71 (100)	19 (100)	14 (88)	13 (93)	6 (86)	2 (100)	7 (64)
Catheter ablation	3 (4)	1 (5)	3 (19)	7 (50)	1 (14)	1 (50)	0
Electrical cardioversion	2 (3)	1 (5)	0	0	1 (14)	0	0
Hospitalization due to arrhythmia	33 (46)	13 (68)	13 (81)	9 (64)	4 (57)	1 (50)	5 (45)
Associated heart disease	51 (72)	13 (68)	8 (50)	2 (14)	2 (29)	2 (100)	9 (82)
Associated other disease (non-cardiac)	31 (44)	10 (53)	5 (31)	1 (7)	2 (29)	2 (100)	6 (55)
NYHA functional class							
I	48 (68)	12 (63)	13 (81)	13 (93)	4 (57)	2 (100)	4 (36)
II	22 (31)	7 (37)	3 (19)	1 (7)	3 (43)	0	7 (64)
III	1 (1)	0	0	0	0	0	0
IV	0	0	0	0	0	0	0
LVEF- mean (SD)	59 (8.93)	55 (14.05)	64(7.78)	71(4.24)	65(5.66)	74 (0)	49 (14.37)
Antiarrhythmic medications in use*							
Class I (%)	2 (3)	0	1 (6)	5 (37)	4 (57)	1 (50)	1 (9)
Class II (%)	57 (80)	18 (95)	9 (56)	2 (14)	2 (29)	1 (50)	5 (45)
Class III (%)	8 (11)	1 (5)	3 (19)	2 (14)	0	0	3 (27)
Class IV (%)	0	0	1 (6)	0	2 (29)	0	3 (27)
Anticoagulant (%)	62 (87)	12 (63)	2 (13)	0	0	1 (50)	0
Know the name of the arrhythmia (%)	11 (15)	0	2 (13)	8 (57)	0	0	1 (9)

* Participants could choose more than one answer option

ASTA-Br-HRQOL scale: The Arrhythmia-Specific Questionnaire in Tachycardia and Arrhythmia—Brazilian version- health-related quality of life scale

AF: Atrial Fibrillation, AFL: Atrial Flutter, AVNRT: *Atrioventricular Nodal Re-entry Tachycardia*, WPW: Wolff-Parkinson-White syndrome, PSVT: Paroxysmal Supraventricular Tachycardia, AT: Atrial Tachycardia, VA: Ventricular Arrhythmia (includes ventricular tachycardia and ventricular extra beats), LVEF: Left Ventricular Ejection Fraction, NYHA: New York Heart Association functional classification, SD: Standard Deviation

### Patient characteristics

There were 87 (62%) patients having an associated cardiovascular disease, 69 (79%) had hypertension, 16 patients (18%) a cardiac valve replacement, 16 (18%) had dyslipidemia, 10 (11%) had Chagas disease, and 2 (3%) a dilated cardiomyopathy. Some patients reported more than one associated cardiovascular disease. As for non-cardiac diseases, the most frequent were diabetes (32–23%) and hypothyroidism (18–13%).

### Construct validity

**Fig 1:**
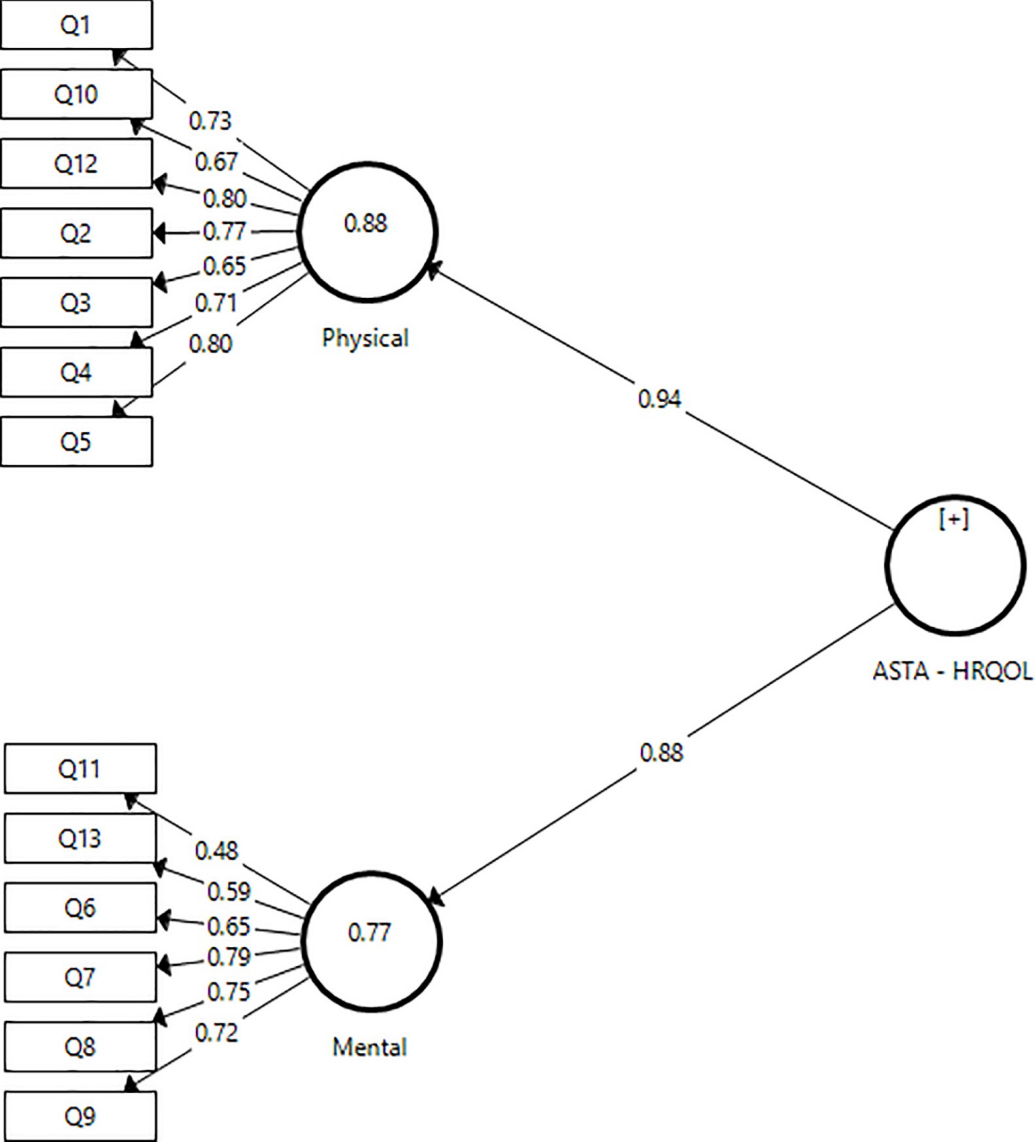
Structural model of the ASTS-Br-HRQOL. Source: SmartPLS 3.2.1.

In order to verify the pre-defined dimensional structure, the construct validity of the ASTA-Br-HRQOL was evaluated by means of a second order CFA. The analysis was performed on the original model of the instrument (Fig 1). The ASTA-Br-HRQOL consists of two domains: one physical and one mental, with 13 questions, of which seven are physically related and six are mentally related.

### Convergent validity of the factor model

In the analysis of the convergent validity of the model, a value higher than 0.5 was observed in the physical domain, but the value was lower in the mental domain (AVE = 0.45). In the mental domain, the item "Q11" presented the lowest factor loading (loaded = 0.48) among the items that compose this domain, characterizing itself as the candidate to be removed from the model. After a qualitative analysis and considering that the value of AVE for the item was just below the recommended minimum of 0.5, it was decided not to exclude the item due to the clinical relevance of this question.

### Discriminant validity of the factor model

The cross-factor loads are presented in Table 4. It can be seen that the items presented higher factor load values in the domains in which they were previously allocated (values in bold), thus indicating the presence of discriminant validity in the factor model.

**Table 4:** Factor loadings of the items in their respective domains.

	Domains of ASTA-Br HRQOL	
	Physical	Mental
**Items**	** **	** **
Q1	**0.73**	0.36
Q2	**0.77**	0.59
Q3	**0.65**	0.45
Q4	**0.71**	0.42
Q5	**0.80**	0.47
Q6	0.46	**0.65**
Q7	0.56	**0.79**
Q8	0.44	**0.75**
Q9	0.48	**0.72**
Q10	**0.67**	0.50
Q11	0.24	**0.48**
Q12	**0.81**	0.57
Q13	0.41	**0.59**

Bold values: factor load values of the items in the domains in which they were previously allocated. ASTA-Br HRQOL = the Arrhythmia-Specific Questionnaire in Tachycardia and Arrhythmia—Brazilian version- health-related quality of life scale.

Table 5 shows the results of the Fornell-Larcker criterion. Since the AVE square root values (values in bold) were higher than the correlation between the constructs, it was concluded that there was discriminant validity in the proposed factor model.

**Table 5:** Square roots of average variance extracted and correlations between the ASTA-Br-HRQOL domains.

Domains	Physical	Mental
**Physical**	**0.74**	---------
**Mental**	0.66	**0.67**

Bold values: square root values of AVE. ASTA-Br HRQOL = the Arrhythmia-Specific Questionnaire in Tachycardia and Arrhythmia—Brazilian version- health-related quality of life scale.

### Convergent validity–correlations between the ASTA-Br-HRQOL and the WHOQOL-BREF domains

Convergent validity was evaluated by correlations of the ASTA-Br-HRQOL physical and mental domain scores and the scores obtained by the WHOQOL-BREF´s respective domain. The correlations obtained were of significant, negative and moderate magnitude (Table 6).

**Table 6:** Spearman’s correlations between ASTA-Br-HRQOL domain scores and WHOQOL-BREF domains.

ASTA-Br-HRQOL	Brazilian WHOQOL-BREF version	
	rho *	p-value
Physical Domain	-0.63	< 0.0001
Mental Domain	-0.58	< 0.0001

*Spearman correlation coefficient

Brazilian WHOQOL-BREF version = the World Health Organization Quality of Life BREF questionnaire. ASTA-Br HRQOL = the Arrhythmia-Specific Questionnaire in Tachycardia and Arrhythmia—Brazilian version- health-related quality of life scale.

### Reliability

The internal consistency of the ASTA-Br-HRQOL scale was evaluated by a composed reliability assessment and Cronbach’s alpha coefficient and considered adequate in both domains (Cronbach’s alpha and composed reliability ≥ 0.70). Table 7 shows the reliability, factor loadings and AVE values for the proposed model.

**Table 7:** Factor loadings, mean extracted value variance, composed reliability and Cronbach’s alpha for the ASTA-Br HRQOL domains.

ASTA-Br-HRQOL Domains	Factor loadings	AVE	Composed reliability	Cronbach´s alpha
Physical	0.65–0.80	0.54	0.89	0.86
Mental	0.48–0.79	0.45	0.83	0.75

ASTA-Br HRQOL = the Arrhythmia-Specific Questionnaire in Tachycardia and Arrhythmia—Brazilian version- health-related quality of life scale.

AVE: Average Variance Extracted.

## Discussion

The aim of this study was to translate and adapt the ASTA-Br-HRQOL scale to make it a suitable tool for assessing HRQOL in patients with different forms of arrhythmia. The main finding of the new Brazilian Portuguese version was that the scale had satisfactory psychometric properties regarding construct validity and internal consistency.

In Brazil, tools available for assessing HRQOL are limited to assessing patients with AF [10, 12]. The ASTA questionnaire was developed and validated with the purpose of evaluating HRQOL in patients with different forms of arrhythmia, i.e., both supraventricular and ventricular [15]. We translated the ASTA questionnaire into a Brazilian Portuguese version which allows comparisons of patients with different arrhythmias and in different cultures and allows Brazilian researchers to compare their data with data from research conducted in other countries that also used the ASTA adapted to their culture. In countries like Brazil, which have a great diversity of cultural roots and many immigrants, cultural adaptation is essential [34]. Even if two-thirds of the patients had no formal education or elementary school certification, there was an understanding and acceptance of the ASTA-Br-HRQOL scale, demonstrated by the interviews, suggesting that the questionnaire is easy to respond regardless education. For those not well educated it is recommended that an interviewer read the questions and the answer options out loud [35].

In the first part using a qualitative approach, we used CVI for evaluating content validity, examining the relevance of the 13 items in the ASTA-Br-HOQOL scale. All except the mentally related item “Do you feel dejected or sad due to your arrhythmia” fulfilled the lower limit of acceptances. Three other items were reviewed by the experts and were somewhat modified. When CVI was assessed in the Swedish version the index for all of the separate HRQOL items and for the total scale was found to be relevant [15].

In the Swedish validation research, CFA was chosen for construct validity to confirm the factor structure of the two pre-defined ingoing domains [15]. The CFA analyses in the present study confirmed the factor model had the strongest factor loadings with the same domains as seen in the Swedish version, i.e., confirmed the pattern found earlier [15]. However, there was one item, “was being afraid of dying due to the arrhythmia”, that did not reach the recommended level of factor loadings in the present study. The finding was discussed by the group of authors and it was decided that even though the value of AVE was lower than the recommended minimum level, the item would be retained due to its clinical relevance. The fear of death and dying is an important existential issue and has resulted in the development of a specific scale assessing this concern, later adapted and validated in Brazil [36, 37]. Using scales aiming to demonstrate impact on a specific domain can be a challenge when the scales indicate that they correlate with each other, i.e., are not independent. This was found for SF-36, where correlations were found between the physical component and mental component summary (PCS/MCS) scores, revealing that PCS measured aspects of mental health and vice versa [38]. It is important to be aware that there are items that can be both physically and mentally related. In research projects, a combination of a disease-specific and a generic questionnaire can be valuable since it enables comparisons with different populations, while a disease-specific questionnaire can be enough for routine clinical use.

Furthermore, the construct was tested against the WHOQOL-BREF questionnaire for convergent validity since both questionnaires contain a physically and a mentally related domain [15, 24, 25]. The WHOQOL-BREF questionnaire has also previously been used along with the ASTA questionnaire, assessing symptoms and HRQOL in patients diagnosed with AF [39]. As expected, the scales in WHOQOL-BREF and ASTA-Br-HRQOL correlated negatively, where higher scores in the ASTA-HRQOL scale reflected an impaired life situation due to arrhythmia, i.e., a more negative impact on HRQOL, while higher WHOQOL-BREF scores indicated the opposite, a better HRQOL.

In the Swedish validation research, the ASTA questionnaire’s HRQOL scale was validated along with SF-36, evaluating convergent and discriminant validity, where the physically related scales correlated significantly as well as the mentally related scales. However, the strongest correlation found in that study was between the two scales in ASTA, revealing that some items can be related to both physical and mental domains [15].

Internal consistency reliability in the ASTA-Br-HRQOL scale was satisfactory for both the physical and mental scales. The original Swedish validation provided similar results and the patient population was the same as in the present study, i.e., people with different forms of arrhythmia. Cronbach’s alpha is sample-dependent and has also demonstrated sufficient reliability (Physical 0,86; Mental 0,75) in a large study evaluating symptoms and HRQOL in patients referred for catheter ablation due to AF, with values above the critical 0.70 level for the two ASTA-HRQOL scales [15, 26, 40].

The psychometric evaluations of the ASTA-Br-HRQOL scale showed similar results to the findings demonstrated in the first Swedish validation study, i.e., satisfactory psychometric properties [15]. By the present study the ASTA-Br-HRQOL scale has been validated and can be used along with the ASTA-Br-Symptom scale [18] for assessments of arrhythmia-related concerns in patients with different forms of arrhythmia. These evaluations can provide important information regarding the patients’ situation and treatment outcomes.

### Methodological considerations/limitations

There are some limitations to be mentioned in the study. Half of the patients included were diagnosed with AF, the overall most common arrhythmia diagnosis, and only a few had atrial tachycardia, paroxysmal atrial tachycardia or ventricular arrhythmia.

Almost 70% of the patients had only an elementary school certification and some had not studied at all and therefore some were not able to read the questions by themselves. A low income was the reality for approximately 80%, which is another way of reflecting the level of education. However, despite the questionnaire being applied in a single center, both the outpatient clinic at the public hospital as well as the private clinic receive patients from different regions of the country and different socioeconomic contexts, which may minimize the need for validation of this instrument in centers throughout the country.

## Conclusions

The newly translated and culturally adapted Brazilian Portuguese version of the ASTA-HRQOL scale, the ASTA-Br-HRQOL scale, was found to have overall satisfactory psychometric properties. The ASTA-Br-HRQOL scale can be a valuable tool for use in research projects as well as for healthcare professionals, preferably in combination with the ASTA-Br-Symptom scale, facilitating the care of patients with different forms of arrhythmia.

## Supporting information

S1 Appendix (DOCX)

S2 Appendix (DOCX)
