# Supplementary material for: A Brazilian Portuguese translation, cultural adaptation and validation of the Arrhythmia-Specific questionnaire in Tachycardia and Arrhythmia (ASTA) health-related quality of life (HRQOL) scale
Source: PLoS One. 2021 Aug 27;16(8):e0256851. doi: 10.1371/journal.pone.0256851 (PMC8396783; doi:10.1371/journal.pone.0256851)
Supplement: S1 Appendix — (DOCX) [file pone.0256851.s001.docx]

**S1 Appendix**

| **ASTA – Health-related quality of life – 13 items** | | **Domain** |
| --- | --- | --- |
| **Question 1** | Do you feel unable to work, study or carry out daily activities as you would like to due to your arrhythmia? | Physical |
| **Question 2** | Do you spend less time with your family/relatives and friends than you would like to due to your arrhythmia? | Physical |
| **Question 3** | Do you spend less time with acquaintances (people you do not know that well) than you would like to due to your arrhythmia? | Physical |
| **Question 4** | Do you avoid planning things you would like to do, for instance traveling or leisure activities, due to your arrhythmia? | Physical |
| **Question 5** | Is your physical ability impaired due to your arrhythmia? | Physical |
| **Question 6** | Is your ability to concentrate impaired due to your arrhythmia? | Mental |
| **Question 7** | Do you feel dejected or sad due to your arrhythmia? | Mental |
| **Question 8** | Do you feel irritated or angry due to your arrhythmia? | Mental |
| **Question 9** | Do you experience sleep problems due to your arrhythmia? | Mental |
| **Question 10** | Is your sexual life affected negatively by your arrhythmia? | Physical |
| **Question 11** | Are you afraid of dying due to your arrhythmia? | Mental |
| **Question 12** | Has your life situation deteriorated due to your arrhythmia? | Physical |
| **Question 13** | Do you feel worried that your symptoms will reoccur during the periods when you do not have arrhythmia? | Mental |

ASTA = the Arrhythmia-Specific Questionnaire in Tachycardia and Arrhythmia
