# Supplementary material for: A Brazilian Portuguese translation, cultural adaptation and validation of the Arrhythmia-Specific questionnaire in Tachycardia and Arrhythmia (ASTA) health-related quality of life (HRQOL) scale
Source: PLoS One. 2021 Aug 27;16(8):e0256851. doi: 10.1371/journal.pone.0256851 (PMC8396783; doi:10.1371/journal.pone.0256851)
Supplement: S2 Appendix — (DOCX) [file pone.0256851.s002.docx]

**S2 Appendix**

| **ASTA-Br- Health-related quality of life – 13 items** | | **Domain** |
| --- | --- | --- |
| **Question 1** | Sua arritmia cardíaca atrapalha você trabalhar, estudar ou realizar atividades diárias como você gostaria? | Physical |
| **Question 2** | Por causa da sua arritmia cardíaca, você passa menos tempo do que gostaria com sua família, parentes e amigos? | Physical |
| **Question 3** | Por causa da sua arritmia cardíaca, você passa menos tempo do que gostaria com conhecidos (pessoas não tão próximas)? | Physical |
| **Question 4** | Por causa da sua arritmia cardíaca você evita fazer planos que normalmente faria, como por exemplo viagens ou outras atividades de lazer? | Physical |
| **Question 5** | Por causa da sua arritmia cardíaca você acha que sua capacidade ou desempenho físico está prejudicado? | Physical |
| **Question 6** | Por causa da sua arritmia cardíaca você acha que sua capacidade de concentração tem piorado? | Mental |
| **Question 7** | Por causa da sua arritmia cardíaca você se sente abatido ou triste? | Mental |
| **Question 8** | Por causa da sua arritmia cardíaca você se sente irritado ou com raiva? | Mental |
| **Question 9** | Por causa da sua arritmia cardíaca você sente que seu sono está prejudicado? | Mental |
| **Question 10** | Por causa da sua arritmia cardíaca você sente que a convivência com seu parceiro(a)/vida sexual está prejudicada? | Physical |
| **Question 11** | Por causa da sua arritmia cardíaca você sente medo de morrer? | Mental |
| **Question 12** | Sua arritmia cardíaca faz com que sua qualidade de vida piore? | Physical |
| **Question 13** | Durante o tempo que você não apresenta arritmia cardíaca, você se sente preocupado que os sintomas possam voltar a aparecer? | Mental |

ASTA-Br-HRQOL = the Arrhythmia-Specific Questionnaire in Tachycardia and Arrhythmia-Brazilian Version
